# Draft Genome Sequence of *Gordonia* sp. Strain YY1, Isolated from an Explosive-Contaminated Environment

**DOI:** 10.1128/MRA.00070-20

**Published:** 2020-04-16

**Authors:** Paula Istvan, Zeev Ronen

**Affiliations:** a Department of Environmental Hydrology and Microbiology, Zuckerberg Institute for Water Research, Jacob Blaustein Institutes for Desert Research, Ben-Gurion University of the Negev, Sede Boqer Campus, Israel; Indiana University, Bloomington

## Abstract

We report the whole-genome sequence of *Gordonia* sp. strain YY1, which was isolated from the surface soil in an explosive-contaminated site in Israel and cultivated with hexahydro-1,3,5-trinitro-1,3,5-triazine, i.e., royal demolition explosive (RDX), as a nitrogen source. This genome sequence will improve our understanding of the genes for RDX degradation. In addition, this research will reveal metabolic pathways in order to develop new bioremediation methods for polluted soil and groundwater.

## ANNOUNCEMENT

Since the extensive use of explosives in World War II, microorganisms have developed mechanisms to degrade them. Cyclic nitramines constitute a group of explosives that contains one or more *N*-nitro groups on heterocyclic rings. An example of this group of explosives is hexahydro-1,3,5-trinitro-1,3,5-triazine, i.e., royal demolition explosive (RDX), which is very commonly used around the world. The toxicity of RDX has raised serious environmental concerns, particularly since the compound is carcinogenic and mutagenic. In addition, it is not highly absorbed by soil particles, which results in its leaching and the formation of groundwater contamination plumes (1, 2). Due to their versatile metabolic properties and genomic and transcriptomic features, members of the genus *Gordonia* are considered to be potentially important for the development of bioremediation-based technologies (3, 4). This genus is included in the mycolic acid-containing order *Actinomycetales,* suborder *Corynebacterineae*, family *Gordoniaceae*, and is well known for utilizing a variety of aliphatic aromatic hydrocarbons and recalcitrant pollutants, including explosives in water and soil (4).

*Gordonia* sp. strain YY1 was isolated from contaminated sandy surface soil at a polluted site located in an industrial zone in the center of Israel, on the eastern outskirts of Ramat Hasharon (5). The enrichment and isolation of strain YY1 were achieved with RDX as a nitrogen source (5). The genomic DNA from a single colony was extracted using the DNeasy blood and tissue kit (Qiagen, Hilden, Germany) and quantified using a Qubit 3.0 fluorometer (Life Technologies, Carlsbad, CA, USA). A genomic DNA library was prepared using the Nextera Flex DNA library preparation kit (Illumina, San Diego, CA, USA). Sequencing was performed using an Illumina MiSeq reagent kit v2 generating 250-bp paired-end reads. A total of 1,849,598 reads were generated by Illumina sequencing. Read quality was assessed with FastQC (version 1.0.0; Illumina BaseSpace Labs). Reads were assembled using SPAdes (version 3.9.0) (6). Coding sequences, mRNAs, rRNAs, tRNAs, genes, and pseudogenes were determined using Prokka (7) and the UniProt database (8). For all software, default parameters were used. The draft genome of *Gordonia* sp. strain YY1 is 5,127,261 bp, distributed in 69 scaffolds. The average coverage was approximately 40×; the *N*_50_ value for the assembly was 22,941.2 bp, and the GC content was 55.83%. The annotation of the *Gordonia* sp. strain YY1 genome identified 4,673 genes, 4,618 coding sequences, 1 transfer mRNA, and 54 tRNAs. This genome will improve our knowledge of RDX degradation genes and metabolic pathways.

### Data availability.

This whole-genome shotgun project has been deposited in DDBJ/ENA/GenBank under accession number WXYI00000000. The raw reads are available in the Sequence Read Archive (SRA) under accession number SRR10846666. The version described in this paper is the first version.
